# Diverticulum of the buccal mucosa: a rare case report and review of the literature

**DOI:** 10.1186/s12903-018-0572-9

**Published:** 2018-06-07

**Authors:** Akihiro Miyazaki, Sho Miyamoto, Hiromi Nakai, Koyo Nishiyama, Kei Tsuchihashi, Jun-ichi Kobayashi, Kazuhiro Ogi, Hironari Dehari, Tadashi Hasegawa, Hiroyoshi Hiratsuka

**Affiliations:** 1 Department of Oral Surgery, Sapporo Medical University School of Medicine, South-1, West-16, Chuo-ku, Sapporo, 060-8543 Japan; 2 Department of Surgical Pathology, Sapporo Medical University School of Medicine, South-1, West-16, Chuo-ku, Sapporo, 060-8543 Japan

**Keywords:** Diverticulum, Oral cavity, Buccinator muscle, Buccal mucosa, Buccal glands

## Abstract

**Background:**

Cases of diverticula of the buccal mucosa are extremely rare. Literature searches of databases such as PubMed/MEDLINE for this condition have revealed only 10 case reports. In this case report, we describe our experience in the management of this rare condition and review the previous 10 previously reported cases.

**Case presentation:**

A 66-year-old man presented with a pouch containing inspissated food debris located posterior to the papilla of the parotid duct in his left buccal mucosa. The diagnosis of a diverticulum arising from the buccal mucosa was confirmed based on clinical and radiographic findings. Gross examination of the locally resected tissue specimen revealed a pouch measuring 14 mm in diameter and 8 mm in depth, that was whitish in color and had an elastic, soft, and smooth surface. Microscopic examination revealed a cyst-like lesion lined by stratified squamous epithelium and granulation tissue, with a chronic inflammatory infiltration in the peripheral stromal tissue of the epithelial layer. After surgical excision of the lesion, there was no recurrence during the follow-up period of 5 years and 10 months.

**Conclusions:**

We have presented a rare case of a diverticulum of the buccal mucosa. This is the first report of a case confirmed not only by the clinicopathological findings, but also by computed tomography and magnetic resonance imaging findings. From the magnetic resonance imaging and intraoperative findings, we inferred that the diverticulum was caused by an idiopathic developmental anomaly due to a partial defect of the buccinator muscle.

## Background

A diverticulum is a protrusion of the inner lining of the digestive tract through a defect in the outer muscular coat to form a small pouch with a narrow neck. The commonest site for development of diverticula is in the lower left portion of the colon. The presence of diverticula is often referred to as diverticulitis, presumably in reference to the irritation caused by retained fecal material [1].

Diverticula of the buccal mucosa are very uncommon. The first report of a diverticulum in the buccal mucosa was described by Bailey [2] as a case of a voluminous diverticulum arising from the buccal sulcus and extending into the neck. Since then, only nine cases have been reported in the literature [3–9].

We herein report a case of a diverticulum of the buccal mucosa, confirmed by clinical examination, computed tomography (CT), and magnetic resonance imaging (MRI), and discuss the etiology of this rare condition.

## Case presentation

**Fig. 1:**
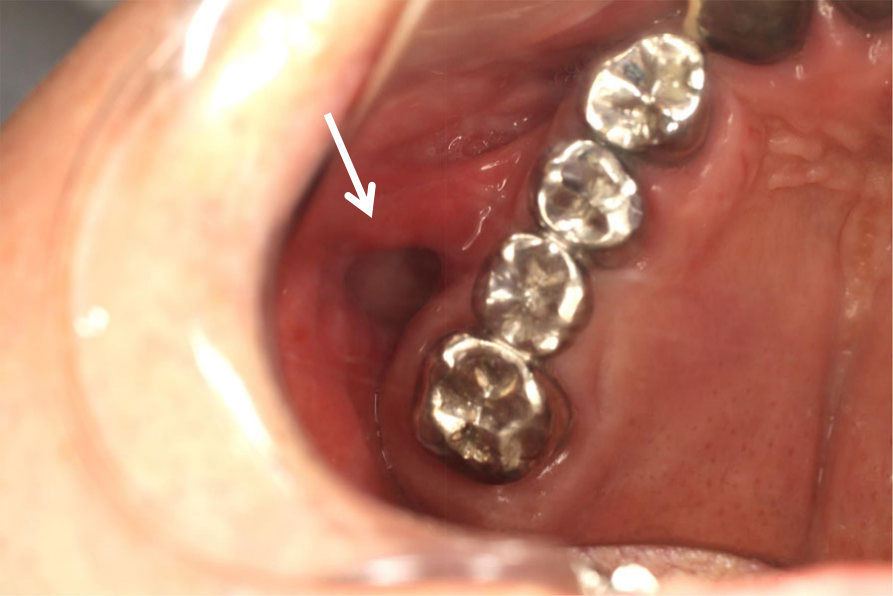
Intraoral view of the buccal mucosa. A pouch in the left buccal mucosa is open, demonstrating a diverticulum (mirror image)

**Fig. 2:**
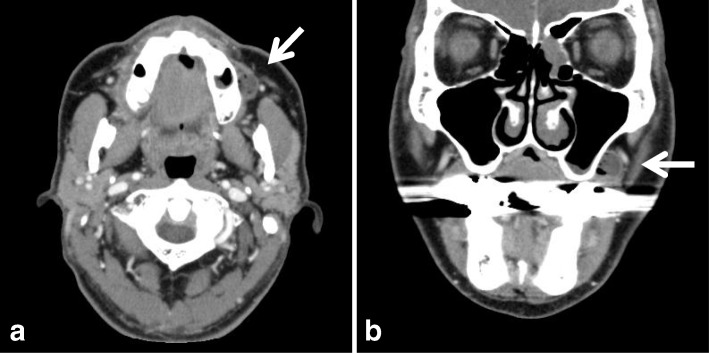
Computed tomography images, a) axial view, b) coronal view, showing a deflection of the buccal mucosa and a cavity containing fluid and air (white arrow)

**Fig. 3:**
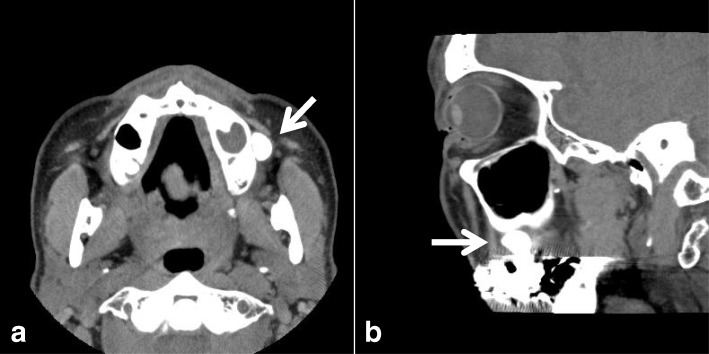
Computed tomography images, a) axial view, b) coronal view, with radiopaque, contrast medium-soaked gauze placed in the cavity which outlines the extent of the pouch (white arrow)

**Fig. 4:**
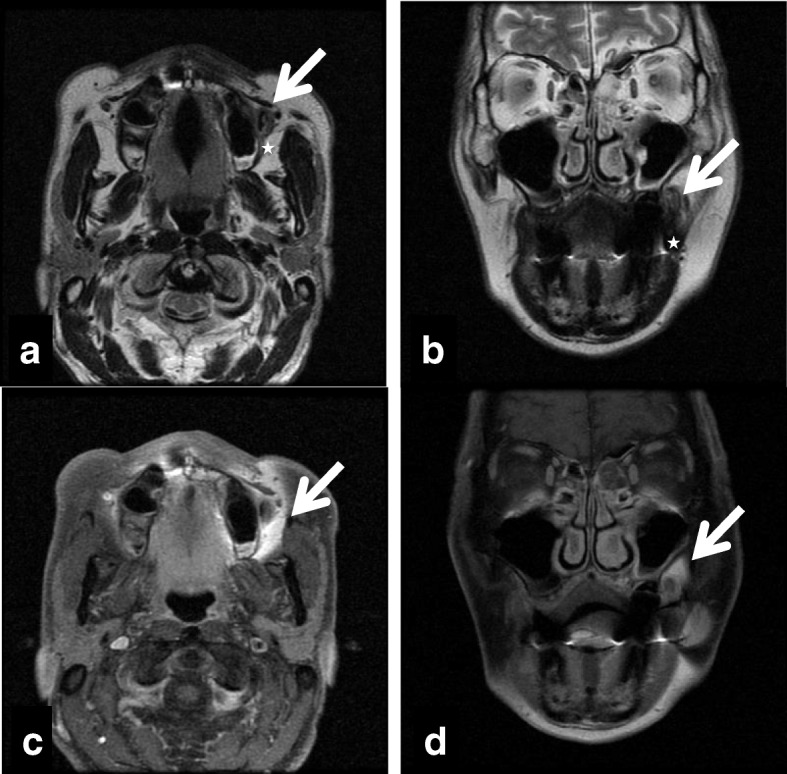
T2-weighted magnetic resonance images, a) axial view, b) coronal view, showing a pouch with an opening and a slightly thickened wall, and a tear in the buccinator muscle on the left side (white arrow). The five-point star indicates the buccinator muscle near the pouch. Gadolinium-enhanced T1-weighted magnetic resonance images, c) axial view, d) coronal view, revealing the wall demonstrating enhancement extending over the surrounding tissues antero-posteriorly in the buccal lesion and superiorly in the maxilla on the left side, which was considered to indicate an inflammatory reaction (white arrow)

A 66-year-old Japanese man was referred to our university hospital in April 2011 with a complaint of food impaction in his left buccal region. The patient had no history of injury, tumor, or cyst in the oral and maxillofacial region. He had a medical history of hypertension, gastric ulcer, gout, nephrolith, and alcohol dependence, but no family history of congenital anomalies. He had been smoking 10–15 cigarettes per day for 45 years. He denied having any allergies. On general physical examination, his physical and nutritional statuses were good. Upon intraoral examination, an orifice containing inspissated food debris was observed posterior to the papilla of the parotid duct in the left buccal mucosa. Neither a mucinous discharge nor a communication with the parotid duct could be observed. A pouch measuring 14 mm in diameter and 8 mm in depth was noted after removing the food debris **(**Fig. 1**)**. A CT examination revealed a discontinuity of his buccal mucosa and a cavity containing fluid and air **(**Fig. 2a, b**)**. An additional CT image with radiopaque, contrast medium-soaked gauze placed in the cavity outlined the extent of the pouch **(**Fig. 3a, b**)**. The MR examinations provided T2-weighted images that showed a pouch with an opening and a slightly thickened wall, with a partial defect in the buccinator muscle on the left side **(**Fig. 4a, b**)**. Gadolinium-enhanced T1-weighted MR images revealed enhancement of the wall extending over the surrounding tissues antero-posteriorly in the buccal lesion and superiorly in the maxilla on the left side, which was considered to indicate an inflammatory reaction **(**Fig. 4c, d**)**. The lesion was diagnosed as a diverticulum of the buccal mucosa and was surgically resected with the patient under general anesthesia. A partial defect in the buccinator muscle was found underneath the pouch during the operation.

**Fig. 5:**
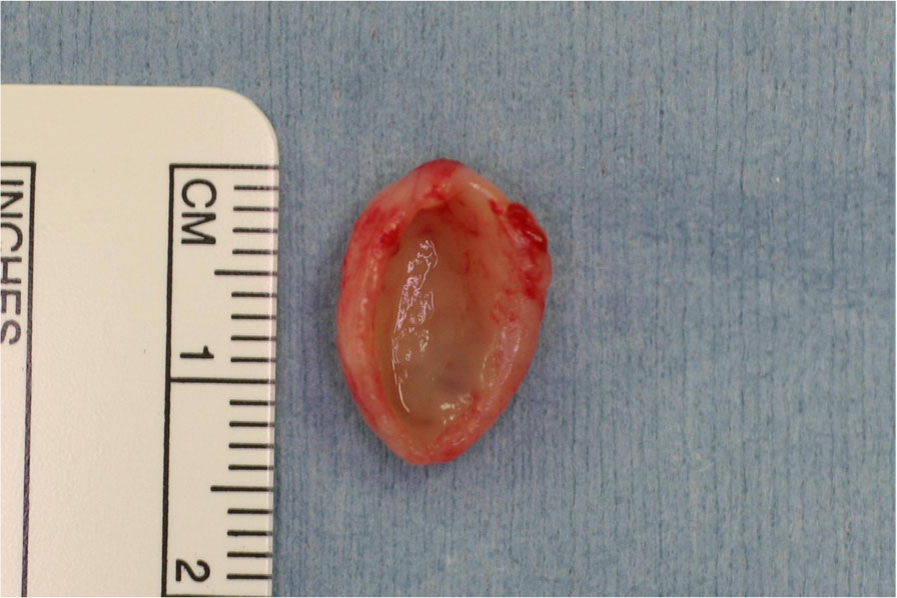
Macroscopic findings. The macroscopic appearance of the excised tissue specimen shows a pouch measuring 14 mm in diameter and 8 mm in depth, that is whitish in color, with an elastic, soft, and smooth surface

**Fig. 6:**
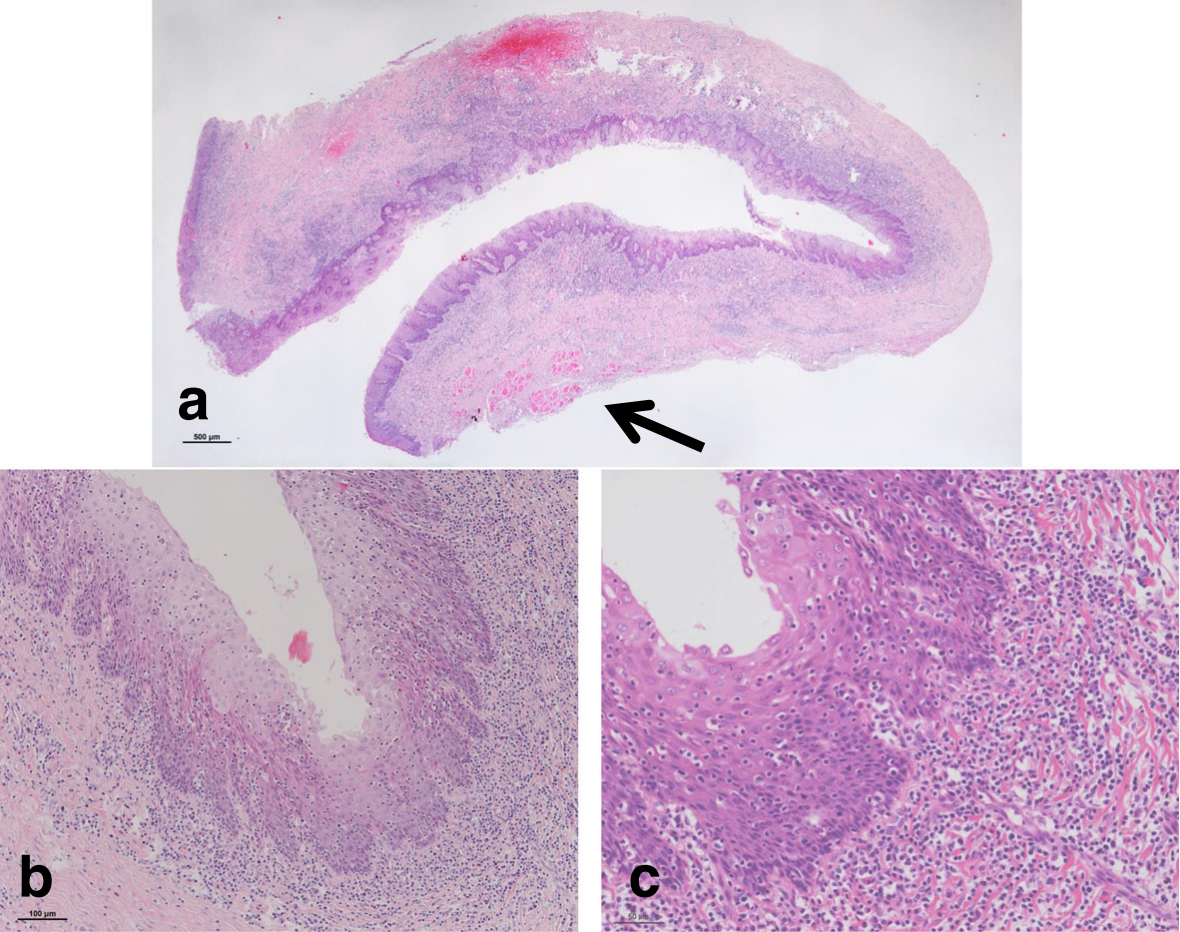
Microscopic findings (with hematoxylin and eosin staining). Histopathological examination of the excised tissue specimen in a) loupe view reveals a pouched appearance with inflammatory infiltration in the stroma. The epithelial lining of the diverticulum is continuous with the surrounding buccal mucosa. The muscle tissue below the lesion is partially observed in the surrounding tissue (black arrow). b) Medium and high magnification (× 100 and × 200) views of the excised specimen show normal buccal epithelium with a collagenous wall and inflammatory cell infiltration

Macroscopically, the excised tissue specimen showed a pouch measuring 14 mm in diameter and 8 mm in depth, that was whitish in color and had an elastic, soft, and smooth surface. **(**Fig. 5**)**. Microscopic examination revealed a cyst-like lesion lined by stratified squamous epithelium and granulation tissue, with chronic inflammatory cell infiltration in the stroma. The affected mucosa was focally depressed and the muscular tissue below the lesion could not be clearly visualized, though the muscle tissue was partially observed in the surrounding tissue **(**Fig. 6**)**.

These findings led to the final diagnosis of a diverticulum of the buccal mucosa. There has been no subsequent food impaction and no evidence of disease recurrence in the 5 years and 10 months since the lesion was resected.

## Discussion and conclusions

A diverticulum refers to the presence of an outpouching in the wall of the digestive tract mucosa, such as the colon or small intestine. The most common type of diverticulum affecting the colon is the pseudodiverticulum, in which the saclike mucosa herniates or projects through the muscularis propria [1]. There have been few reports of diverticular disease in the oral cavity. However, it is possible that some cases were not reported due to an incorrect diagnosis, asymptomatic clinical course, presence of few symptoms, or low morbidity of the lesion.

The patient characteristics from the 11 reported cases of buccal diverticula, including our case, are presented in Table 1. There was an obvious sex predilection, with nine patients being male and only two being female. The age range for all reported cases was 42–89 years (mean age, 68 years) and there was a predisposition for elderly individuals. Four cases had two pouches, bilateral or unilateral, and seven cases had one pouch. The reports of this lesion are predominant in Japan with eight patients, and the remaining 3 cases comprise one from Taiwan and two from the United Kingdom. Although nine cases actually belong to the East Asian nations, the influence of the ethnicity is unclear and further case presentations from other nations are expected to elucidate this aspect.

**Table 1:** Summary of the patient characteristics in 11 cases of diverticular lesions of the buccal mucosa

Patient	Age (years)/Sex	Chief complaint	Side of the buccal mucosa	Location within the buccal mucosa	Diameter (mm)	Depth (mm)	Treatment	Nation	Reference
1	86/F	Swelling	Right	Inferior	30	100	Excision	United Kingdom	Bailey [2]
2	63/M	Bad breath	Left	Posterior-inferior	15	11	Follow up	Japan	Takeda [3]
3	46/M	Food impaction	Right	Inferior	21	13	Excision	Japan	Yamamoto et al. [4]
4	89/F	Symptomless	Right	Anterior	Not reported	Not reported	Follow up	United Kingdom	Rowson [5]
			Right	Posterior	Not reported	Not reported	Follow up		
5	85/M	Food impaction	Bilateral	Posterior-inferior	7	7	Excision	Japan	Kubo et al. [6]
		Food impaction		Posterior-inferior	3	Not reported	Follow up		
6	80/M	Symptomless	Bilateral	Posterior	10	10	Follow up	Taiwan	Yu [7]
				Posterior	10	6	Follow up		
7	52/M	Symptomless	Right	Posterior-inferior	3	7	Excision	Japan	Terada et al. [8]
8	80/M	Symptomless	Right	Inferior	12	5	Follow up	Japan	Terada et al. [8]
9	42/M	Symptomless	Right	Inferior	3	8	Follow up	Japan	Terada et al. [8]
10	60/M	Food impaction	Right	Inferior	5	10	Excision	Japan	Ohnuki et al. [9]
			Right	Inferior	3	5	Excision		
11	66/M	Food impaction	Left	Posterior	13	8	Excision	Japan	Present case

The etiology of the buccal diverticulum is unclear, though some theories have been reported. First, the cheek is anatomically composed principally of the buccinator muscle, which consists of three groups of muscle fibers [10]. It has been proposed that a diverticular lesion is caused by a developmental anomaly occurring due to partial dissection along those muscle fibers [3, 4]. Second, Rowson [5] postulated that chronic food inspissation causes a small traumatic lesion, which might progress into the formation of a diverticulum over time due to thin, atrophic mucosa and poor muscle tone. Kubo et al. [6] also suggested the possibility of a relationship with chronic food inspissation due to the disease’s prevalence in the elderly, in whom poor oral hygiene is common. Third, Bailey [2] postulated that diverticulum formation may result from abnormal salivary tissue growth. Hypothetically, a salivary gland neoplasm arising from aberrant salivary tissue may become necrotic, and subsequently form a diverticulum in the normal squamous buccal mucosa. Fourth, it has been suggested that diverticula of the buccal mucosa may be due to idiopathic developmental defects resulting from invagination of the primary epithelial band, or may represent aborted development of accessory parotid primordial invaginations [7] or that of buccal glands [9]. The etiology remains unclear because sufficient evidence supporting any of these theories has not been collected. In our case, the diverticulum appeared to be an idiopathic developmental anomaly. Furthermore, a partial defect in the buccinator muscle fibers was observed on microscopic examination. The above stated evidence suggests that the type of diverticulum affecting the buccal mucosa was a pseudodiverticulum resulting from a defect in the buccinator muscle. In our case, dermal or epidermal inclusion cyst and fistulous tract could be excluded from the differential diagnosis since the morphology was that of a pouch but not a cystic or ductal structure. Furthermore, because of lack of keratinizing stratified squamous epithelium lining and keratin in the lumen or remarkable inflammatory granulation tissue and abscess, the diagnosis was supported from the histopathological aspect.

With respect to clinical manifestation, the diverticula appeared as asymptomatic lesions in five of the reported cases, while the other cases presented with halitosis, food impaction, or swelling. Surgical treatment should be recommended in symptomatic cases involving food impaction or voluminous diverticulum. Of the 11 reported cases, six cases underwent surgical excision and the remaining five cases received conservative treatment, including periodic irrigation and cleansing of the oral cavity. Since our patient complained of food impaction, local excision was the most appropriate treatment option. After the surgery, the patient’s symptom of food impaction resolved and there has been no recurrence to date.

In conclusion, this report has described a rare occurrence of a diverticulum of the buccal mucosa. While a small number of other reports of this condition do exist, this is the first proven case, and we have provided substantial evidence in the form of clinical, pathological, and radiographic findings. In addition, we were able to elucidate that an idiopathic developmental anomaly caused by a buccinator muscle defect was responsible for the development of the diverticulum in this case.

## Abbreviations

CT: computed tomography
MRI: magnetic resonance imaging
